# Editorial: Abdominal aortic aneurysms: advancements in diagnosis, biomarkers, drug therapeutics, surgical and endovascular treatment

**DOI:** 10.3389/fcvm.2023.1218335

**Published:** 2023-06-02

**Authors:** Zhenjie Liu

**Affiliations:** Department of Vascular Surgery, The Second Hospital of Zhejiang University School of Medicine, Hangzhou, China

**Keywords:** abdominal aortic aneurysm, diagnose, biomarker, drug therapeutic, surgical and endovascular treatment

**Editorial on the Research Topic** Abdominal aortic aneurysms: advancements in diagnosis, biomarkers, drug therapeutics, surgical and endovascular treatment

An abdominal aortic aneurysm (AAA) is a dilated aorta with the diameter 1.5 times the normal one, involving all three anatomical layers. AAAs mostly remain asymptomatic until the aneurysm ruptures, which can be unpredictable and fatal, with a mortality rate of over 50% (1). Current guidelines recommend watchful waiting and lifestyle adjustment for smaller, slow-growing aneurysms, while elective/prophylactic surgical repairs are recommended for larger aneurysms that have grown beyond certain thresholds (55 mm for males and 50 mm for females) (2, 3).

Considerable advancements have been made in the management of AAA, specifically in endovascular treatment, through the development of novel stent grafts and synthetic grafts that cater to intricate and challenging aortic anatomies. Unfortunately, there are no effective medical therapies for AAA, especially for newly diagnosed patients who do not have surgical indications (4). Despite the fact that many drugs, such as Doxycycline and Rapamycin, demonstrated efficacy in animal models, the outcomes of clinical trials are disappointing (5–7). For asymptomatic AAAs, aortic ruptures are life-threatening. Early detection for impending rupture of AAA is still inaccessible.

An increasing number of researchers are dedicating their efforts to studying AAA, resulting in significant progress in the field. In this research topic, an international selection of researchers contributed original research, case reports, and up-to-date reviews to enhance our current understanding of AAA. These studies have focused on various aspects of this disease, including improved methods of diagnosis, predictable biomarkers, drug therapeutics, and surgical and endovascular treatments.

## 1. Basic research works

Several contributions in this research topic focused on identifying biomarkers and potential treatment options. Lowis et al. reviewed the mechanosignals involved in AAA development, including divergent mechanosignal, endothelial, and smooth muscle cell mechanosensors, mechanical stress, inflammation, and redox stress circuits. They also briefly reviewed intraluminal thrombus and mechanosensing in AAA, highlighting the manipulation of mechano-machinery as a potential avenue for future research in AAA. Another review article discussed the roles of extracellular vesicles (EV) in the pathological process of AAA and their potential clinical applications. Lu et al. reviewed the EV-based biomarkers for AAA diagnosis and the therapeutic potential of stem cell-derived EVs. With advancements in EV display technology and membrane hybrid technology, researchers have been able to equip EVs with desirable functions, making these vesicles a promising tool for the treatment of AAA.

While some researchers reviewed biomarkers in AAA, others have focused on gene expression changes as a potential avenue for understanding the development and progression of AAA. Chen et al. conducted a study that focused on immune infiltration patterns in AAA by constructing a competing endogenous RNA network. They obtained expression profiles of circRNAs and mRNAs from Gene Expression Omnibus (GEO) to build a ceRNA network and validated key genes expressions and immune cell infiltration using clinical specimens. Their study identified significant roles of PPARG, FOXO1, RAB5C, and HSPA8, as well as the involvement of M1 macrophages and resting CD4 memory T cells. Another work by Chen et al. identified the biomarkers and analyzed infiltrated immune cells in stable and ruptured abdominal aortic aneurysms. Unlike the result in AAA vs. healthy aorta, this study showed that NFKB1 might be an important transcription factor mediating the inflammatory response of AAA and aortic rupture, and CD19, SELL, and CCR7 were selected as hub genes for AAA, whereas OAS3, IFIT1, and IFI44l were identified as hub genes for aortic rupture in a predictive model constructed. However, experimental evidence is still necessary since the result varies from databases and different statistical methods.

Animal models play a crucial role in investigating the pathophysiology of AAA and developing new therapeutic and diagnostic approaches. So far, the three most widely adopted AAA-inducing methods are angiotensin II (AngII) infusion (8), porcine pancreatic elastase intraluminal perfusion (9), and calcium salt topical application (10), featuring varying incidence rates, anatomical locations, lesion sizes, and disease courses. With the emerging interest and research efforts dedicated to the propagation phase of AAA, it is of significant mechanistic and translational value to identify the appropriate experimental models that recapitulate the progressive AAA progression into rupture (11). Yin et al. reviewed the progress in murine models of RAAA. The ideal RAAA model should provide consistent aneurysm formation in the appropriate anatomical location (i.e., infrarenal aortic segment) and consistent AAA rupture over a chronic course. Although few of the existing models could faithfully recapitulate the clinical features of RAAA, some models, such as β-aminopropionitrile combined with topical application of elastase, and AngII combined with elastase intraluminal perfusion model, have the potential to be modified to an RAAA model.

In addition to reviews, original research studies are published on this research topic. Griepke et al. demonstrated the effectiveness of selectively inhibiting soluble tumor necrosis factor signaling in reducing abdominal aortic aneurysm progression in a rat AAA model. Chinese researchers shared their study which specially focused on traditional Chinese medicine therapy in cardiovascular diseases. Hong et al. found that Xin'an famous prescription Ershen Zhenwu Decoction (ESZWD) significantly reduced left ventricular end-diastolic diameter, decreased N-terminal pro-brain natriuretic peptide (NT-proBNP), angiotensinII, aldosterone, reactive oxygen species, and malondialdehyde, and increased serum superoxide dismutase, without significantly affecting inflammatory factors. They investigated thirty components of ESZWD and identified salsolinol, aconitine, paeoniflorin, and miltrione as the potential pharmacodynamic substances for ESZWD in treating CHF-HKYd. This work suggests a potential application of traditional Chinese medicine therapy in other cardiovascular diseases, including AAA.

## 2. Laboratory and CTA assisted diagnose

In addition to their foundational research endeavors, colloquially known as bench work, several clinical scientists presented their research projects based on clinical-oriented understandings.

Cai et al. compared the laboratory test results of 320 patients with peripheral artery diseases and 320 patients with abdominal aortic aneurysms. They found that levels of bilirubin and D-dimer increased in AAA-only patients compared to PAD-only patients (*P* < 0.001). Further analysis confirmed a differential distribution of bilirubin, D-dimer, fibrinogen, and platelet count between AAA patients and PAD patients (*P* < 0.05), and suggested a cutoff value of 0.675 mg/L for D-dimer, which could be used to predict AAA in PAD patients.

Computed tomography (CT) scan is an essential diagnostic tool in AAA patients. Liu et al. attempted to measure the maximum transverse diameter (MTD) of and CT values of ILT by using multi-spiral computed tomography angiography (MSCTA) to investigate the predictive value of MTD with different CT values of thrombus on the risk of AAA rupture. Their study 45 intact AAA and 17 ruptured AAA MSCTA results. The median of maximum CT value of thrombus at the plane of MTD was higher in RAAA (107.0 HU) than the median in IAAA (84.5 HU) (*P* < 0.001). The maximum CT value was also a significant risk factor for RAAA (*P* < 0.001). High-density ILT shown on MSCTA in AAAs was associated with aneurysm rupture, and its maximum transverse diameter combined with the maximum CT value in its plane is a better predictor of RAAA.

To diagnose AAA using CT, it's important to have appropriate reference values for a given population. Therefore, it is important to establish reference values specific to the population being studied to ensure accurate and reliable diagnosis. A study by Wang et al. provided valuable information on the size of normal abdominal aortas in different segments among Chinese adults (>18 years) without abdominal aortic disease, using enhanced CT. The study measured the inner-to-inner areas of the subphrenic, suprarenal, infrarenal, and distal segments of the abdominal aorta in 1,066 patients. The median areas of the subphrenic abdominal aorta, suprarenal abdominal aorta, infrarenal abdominal aorta, and distal abdominal aorta were 412.1 mm^2^, 308.0 mm^2^, 242.2 mm^2^ and 202.2 mm^2^ in Chinese male population, and the median areas of the subphrenic abdominal aorta, suprarenal abdominal aorta, infrarenal abdominal aorta, and distal abdominal aorta were 327.7 mm^2^, 243.4 mm^2^, 185.4 mm^2^ and 159.6 mm^2^ in female Chinese population. These reference values can be useful in the diagnosis and management of abdominal aortic diseases in the Chinese population.

## 3. Research focused on non-surgical management of AAA

The management of AAA poses a challenge when dealing with smaller aneurysms without surgical indications as there is no established way to slow down the progression of the aneurysm's dilation (4). The management of AAA poses a challenge when dealing with smaller aneurysms that do not require surgery, as there is currently no established approach to impede the progression of their dilation. Consequently, numerous researchers are committed to discovering effective techniques and medications to enhance the current watchful observation strategy for small aneurysms.

A comprehensive understanding of the natural history of AAA is crucial for developing effective ways to slow down the dilation of the aorta. To this end, Wu et al. conducted a prospective cohort study on the natural history of isolated abdominal aortic dissection, which shared some similar pathophysiological changes with AAA. The study included 68 patients who were followed up for up to 5.5 years. The findings suggested a more aggressive treatment regimen for IAAD, with EVAR being the first choice, especially for those with persistent symptoms and patent false lumen, regardless of sex, age, or aortic size. This study may indicate a more aggressive treatment strategy for AAA with dissection.

In recent years, there has been a growing interest in lifestyle changes as a means of managing small abdominal aortic aneurysms (AAA), in light of the “watchful and wait” strategy. Although smoking cessation and physical exercise both have shown promising benefits in reducing AAA risks, few studies concerned the role of healthy dietary patterns, at both clinical and preclinical levels (12–14). With an increasing benefits of various dietary regimens against cardiovascular diseases recently, understanding the therapeutic and mechanistic implications of certain dietary patterns would hold significant value in informing the future guideline of AAA management (15). Herein, Yin et al. summarized the recent progress in dietary therapies for AAA based on epidemiological and experimental evidence. Additionally, they discussed perspectives of emerging dietary regimens and potential molecular basis. Further investigations are still pending in selected emerging dietary regimens as well as diet-mediated mechanisms. And it can be envisioned that future research efforts will be increasingly dedicated to a better understanding of as well as translational development of the first non-surgical management of AAA in the form of dietary therapies.

Despite growing interest in lifestyle interventions for AAA management, pharmacological approaches remain more commonly explored. Niu et al. conducted a meta-analysis reviewing the association between metformin and abdominal aortic aneurysm, which include 10 cohort studies and 85,050 patients. The findings suggest metformin may limit the expansion of AAA and reduce the incidence of AAA and postoperative mortality. However, given the limited number of trials included, further biological experiments and clinical trials are still necessary to validate these results and establish metformin's role in AAA management.

Besides the potential benefits of drug therapies, the effects of different drugs on AAA treatment and adverse outcomes remain a critical area of research. A review by Chen et al. focused on the role of fluoroquinolones (FQs) in aortic aneurysms and dissections incidence and mortality. They summarized relevant clinical trials and found that FQs were associated with an increased incidence of AAD in the general population and a higher risk of adverse outcomes in patients with pre-existing AAD. Although the authors noted that the results may be affected by unmeasured confounding factors, it should be considered by physicians contemplating using FQs in patients with aortic dilation and those at high risk of AAD.

## 4. Surgical and endovascular treatment

Endovascular aneurysm repair (EVAR) has been established as a viable alternative to open repair surgery for treating AAA (2, 3). However, secondary aneurysm ruptured caused by endoleaks and a high rate of secondary intervention limite EVAR's survival benefit (16, 17). Type II endoleak (T2EL) is the most common type of endoleaks (18, 19). To address this issue, several strategies for pre-emptive embolization have been developed, including embolization of aneurysm sac side branches (ASSB) and aneurysm sac coil embolization (ASCE) (20–22). Wu et al. conducted a network meta-analysis to compare the effectiveness of different preventive interventions for T2EL in the suppression of aneurysm sac expansion and reducing the need for re-intervention with 31 studies involving 18,542 patients. The study found that all prophylactic embolization strategies improved the clinical outcomes of EVAR, with pre-emptive embolization of the inferior mesenteric artery (IMA-ASSB) showing the best clinical effect in suppressing aneurysm sac expansion and reducing the need for re-intervention, while non-selective embolization of ASSB (NS-ASSB) was found to be more effective in reducing the incidence of T2EL. IMA-ASSB alone or in combination with ASCE demonstrated benefits in clinical outcomes of EVAR. However, it should be noted that network meta-analysis is still an indirect method based on existing data, and more studies are needed to establish more credible conclusions.

In addition to endoleaks, other patient-related factors can impact the outcomes of EVAR. Wang et al. developed and compared multi-modal models based on morphological, deep learning (DL) and radiomics features to predict outcomes after EVAR. The result showed that the radiomic model based on logistics regression had a better predictive performance for patient status after EVAR than the optimized morphological feature model and the DL model. The morphological feature model and DL model have their own advantages and could also be used to predict outcomes after EVAR. These findings provide valuable insights into EVAR treatment and post-operation follow-up.

RAAAs, known as the life-threatening complication of AAA, have up to an 85% mortality rate (23). Ruptured endovascular aneurysm repair (rEVAR) is now considered as an alternative to operative treatment for RAAAs (2, 3). Meanwhile, the discussion about the role of rEVAR has been sustained during the past two decades due to the discrepancy between randomized controlled trials (RCTs) and real-life studies (24–27). In the retrospective study conducted by Fang et al., the efficacy and safety of EVAR-selected and EVAR-only strategies in the management of RAAA were evaluated. Results showed no difference in third-day mortality and long-term outcomes between the EVAR-only strategy and the EVAR-selected strategy, while the EVAR-only strategy was associated with a more simplified algorithm, less influence on hemodynamics, and a shorter operation and recovery time, indicating a non-inferior feasibility of EVAR-only strategy to EVAR-selected strategy.

The surgical strategy for infected abdominal aortic aneurysms poses another significant challenge. Li et al. reported their cases about *in situ* repair or reconstruction of the abdominal aorta-iliac artery using autologous fascia-peritoneum with posterior rectus sheath for the treatment of the infected abdominal aortic and iliac artery aneurysms. They repaired the aneurysms using autologous peritoneal fascial tissue, supplemented with grafts when necessary. After 2–19 months follow-up, five out of seven patients survived. While long-term follow-up and a larger sample size are necessary to establish the efficacy of this treatment, the *in situ* repair or reconstruction with autologous peritoneal fascial tissue with rectus sheath appears to be a promising and feasible option for infected aneurysm patients without adequate autologous venous substitutes.

In addition to studies focused directly on AAA, we also collect researches in other related areas that may also contribute to the management of AAA. Huang et al. designed a modular multifunctional left heart bypass (LHB) circuit integrated with ultrafiltration and reserved pipelines for cardiopulmonary bypass in thoracoabdominal aortic aneurysm repair. This circuit made the assembly of the LHB circuit more easily, and more efficient, which may contribute to the TAAA repair operation performed in lower volume centers easily. Another study by Luo et al. provided us with some concerns about in-hospital care for AAA patients. Their study highlighted the significance of elevated blood creatinine and urea nitrogen levels at ICU admission and their correlation with the risk of in-hospital death and 1-year mortality in patients with intracranial hemorrhage. This is especially relevant for elderly AAA patients who are at higher risk of intracranial hemorrhage. By monitoring these biomarkers and providing timely interventions, healthcare providers can potentially improve patient outcomes and reduce mortality rates. This study highlights the importance of comprehensive and personalized care for AAA patients, which takes into account individual risk factors and biomarkers.

In conclusion, the diverse and high-quality contributions of this Research Topic enriched our understanding of diagnosis, biomarkers, drug therapeutics, surgical and endovascular treatment in AAA. Moreover, these studies have opened up potential avenues for further research in this field, while also providing novel insights that may lead to improved management and outcomes for patients with AAA.
